# Tg2576 Cortical Neurons That Express Human Ab Are Susceptible to Extracellular Aβ-Induced, K^+^ Efflux Dependent Neurodegeneration

**DOI:** 10.1371/journal.pone.0019026

**Published:** 2011-04-27

**Authors:** Shannon Ray, Claire Howells, Emma D. Eaton, Chris W. Butler, Lana Shabala, Paul A. Adlard, Adrian K. West, William R. Bennett, Gilles J. Guillemin, Roger S. Chung

**Affiliations:** 1 NeuroRepair Group, Menzies Research Institute, University of Tasmania, Hobart, Tasmania, Australia; 2 Synaptic Neurobiology Lab, Mental Health Research Institute, Melbourne, Victoria, Australia; 3 Neuroinflammation Group, University of New South Wales, Sydney, New South Wales, Australia; Emory University, United States of America

## Abstract

**Background:**

One of the key pathological features of AD is the formation of insoluble amyloid plaques. The major constituent of these extracellular plaques is the beta-amyloid peptide (Aβ), although Aβ is also found to accumulate intraneuronally in AD. Due to the slowly progressive nature of the disease, it is likely that neurons are exposed to sublethal concentrations of both intracellular and extracellular Aβ for extended periods of time.

**Results:**

In this study, we report that daily exposure to a sublethal concentration of Aβ_1-40_ (1 µM) for six days induces substantial apoptosis of cortical neurons cultured from Tg2576 mice (which express substantial but sublethal levels of intracellular Aβ). Notably, untreated Tg2576 neurons of similar age did not display any signs of apoptosis, indicating that the level of intracellular Aβ present in these neurons was not the cause of toxicity. Furthermore, wildtype neurons did not become apoptotic under the same chronic Aβ_1-40_ treatment. We found that this apoptosis was linked to Tg2576 neurons being unable to maintain K^+^ homeostasis following Aβ treatment. Furthermore, blocking K^+^ efflux protected Tg2576 neurons from Aβ-induced neurotoxicity. Interestingly, chronic exposure to 1 µM Aβ_1-40_ caused the generation of axonal swellings in Tg2576 neurons that contained dense concentrations of hyperphosphorylated tau. These were not observed in wildtype neurons under the same treatment conditions.

**Conclusions:**

Our data suggest that when neurons are chronically exposed to sublethal levels of both intra- and extra-cellular Aβ, this causes a K^+^-dependent neurodegeneration that has pathological characteristics similar to AD.

## Introduction

Alzheimer's disease (AD) is characterised by profound synaptic loss and neuronal death, and the accumulation of a number of key pathological hallmarks; senile plaques, dystrophic neurites and neurofibrillary tangles [1]. The β-amyloid peptide (Aβ) is the principle component of plaques, and is thought to contribute significantly to the pathogenesis of the disease [2]. However, the precise mechanisms that underlie the role of Aβ in AD are not clearly understood.

The localisation of Aβ is likely to have an important role in governing its toxic actions upon neurons. In this regard, it is well known that acute extracellular administration of aggregated forms of Aβ (and in particular oligomers) to cultured neurons is neurotoxic [3]. This is in accordance with the presence of amyloid plaques in AD, of which extracellular, aggregated forms of Aβ are the major constituent [4]. However, a growing body of evidence suggests that intraneuronal localisation of Aβ may also play a significant role in AD. For example, Aβ accumulates in processes and synapses prior to, and with the onset of extracellular Aβ plaque formation [5], [6], and in transgenic mice that develop Aβ plaques [7]. There is also some evidence that cognitive impairment in AD patients does not always correlate to the level of Aβ plaque deposition [8]. Similarly Aβ immunisation studies in Tg2576 [9] or PDAPP [10] transgenic mice reversed memory loss, but had no impact upon amyloid plaque levels. These studies suggest that the intraneuronal accumulation of Aβ may be important in disease progression and symptom onset.

Indeed, intracellular Aβ appears to increase the susceptibility of neurons to neurodegeneration. For example, Abdul *et al* [11] reported that cortical neurons cultured from APP/PS1 transgenic mice were more vulnerable to oxidative stress, mitochondrial dysfunction and apoptosis. Yao and colleagues [12] have demonstrated that 3xTg-AD mice exhibit increased hydrogen peroxide production and lipid peroxidation. Furthermore, hippocampal neurons cultured from these mice exhibited significantly decreased mitochondrial respiration and increased glycolosis [12]. Notably, cultured neurons transfected with constructs expressing APP that contains familial-linked AD mutations that substantially increase levels of Aβ are also susceptible to apoptosis-inducing treatments [13]. Others have linked intracellular Aβ directly to apoptosis, reporting that transfection of constructs expressing Aβ into neuroblastoma cells resulted in activation of a P53-dependent apoptotic pathway [14]. A similar outcome has been observed when synthetic Aβ peptides were microinjected into neurons, which induced cytotoxicity via a p53-Bax apoptotic pathway [15]. Notably, exogenously applied Aβ is rapidly internalised by cultured neurons, where it could subsequently act in a neurotoxic manner like endogenous Aβ. Uptake of Aβ by neuronal cells occurs via the low-density lipoprotein receptor LRP1 [16]. Treatment of PC12 cells [17] or primary neuron cultures [18] with exogenous Aβ leads to accumulation of reactive oxygen species, and a decrease in redox activity and ATP levels [17]. Treatment with alpha-tocopherol (Vitamin E) can block these Aβ-induced changes in neuronal cells (see reviews by [19], [20]).

AD is a progressive disease, in which neurons are likely to be exposed to both intracellular and extracellular Aβ at sublethal concentrations for extended periods of time. To experimentally model this situation, we have exposed cultured neurons from Tg2576 mice (which accumulate substantial amounts of intracellular Aβ) to daily treatment with exogenous Aβ_1-40_ for 6 days. Chronic Aβ_1-40_ treatment induced substantial apoptosis of cortical Tg2576 neurons but not wildtype neurons, suggesting that both intra- and extra-cellular Aβ are required to induce apoptosis. Apoptosis was linked to an inability of Tg2576 neurons to maintain K^+^ homeostasis following acute treatment with extracellular Aβ_1-40_. Chronic exposure to 1 µM Aβ_1-40_ also caused the generation of hyperphosphorylated tau-immunoreactive axonal swellings in Tg2576 but not wildtype neurons. Our data suggest that chronic exposure to both intra- and extra-cellular Aβ induces neurodegenerative changes that bear similarities to some of the pathological hallmarks of AD.

## Results

### Uptake of exogenous Aβ by cultured transgenic Tg2576 and wildtype cortical neurons

**Figure 1:**
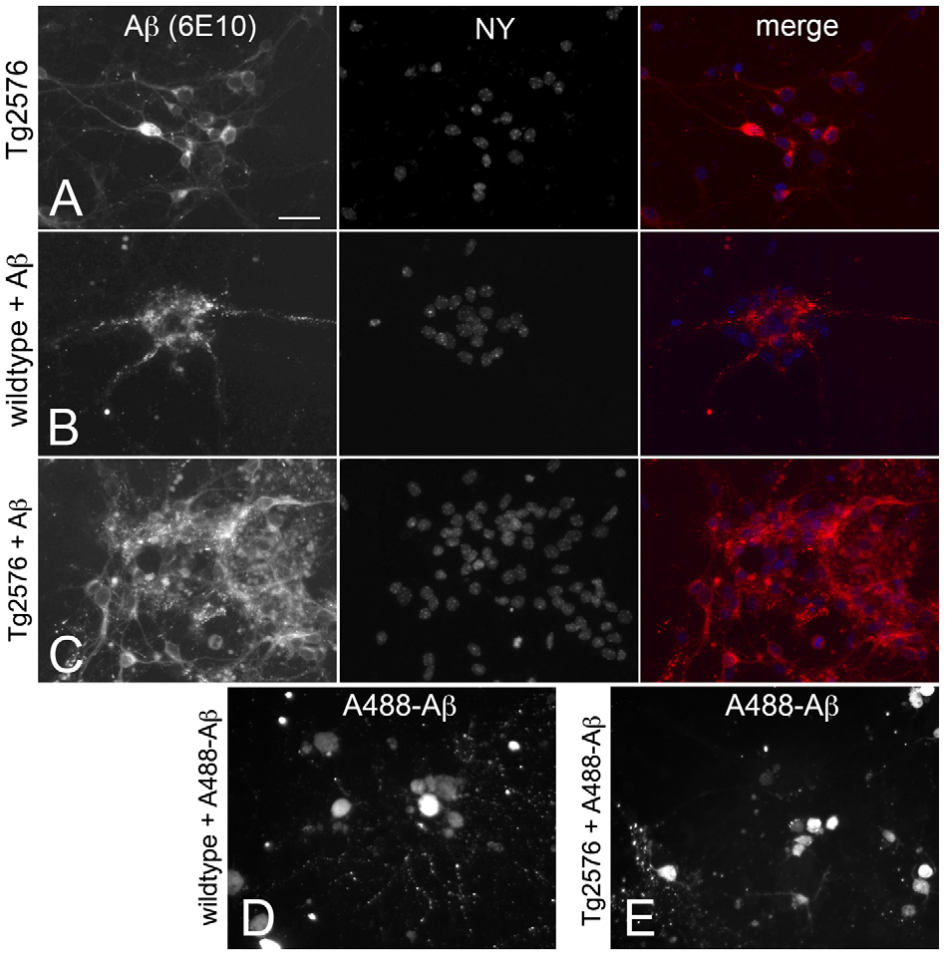
Uptake of soluble Aβ by wildtype and Tg2576 cortical neurons *in vitro*. Wildtype and Tg2576 cortical neurons were treated with 10 µM of monomeric Aβ_1-40_, and immunostained for Aβ after 24 hours. In untreated Tg2576 neurons, Aβ was smoothly distributed throughout the cytoplasm and processes of all neurons (A). When Aβ_1-40_ was applied to wildtype neurons, it was internalised and distributed in a punctate manner within the cytoplasm and processes (B). Notably, not all wildtype neurons internalised Aβ_1-40_ (B). When Aβ_1-40_ was applied to Tg2576 neurons, both smooth and punctately distributed Aβ was detected within neurons (C). scale bar  = 25 µm.

Mouse cortical neurons (wildtype and Tg2576) were maintained in culture for seven days *in vitro* (DIV), at which time they were treated with 10 µM soluble monomeric Aβ_1-40_. After 24 hours, neurons were fixed and Aβ detected by immunostaining. In untreated Tg2576 cortical neurons, Aβ was distributed within the cytoplasm and processes, but generally not in the nucleus (Figure 1A). In untreated wildtype cortical neurons, there was no Aβ detected (results not shown). In wildtype neurons treated with Aβ, Aβ was detected in a punctate distribution within the cytoplasm and processes (Figure 1B). When Aβ was applied to Tg2576 neurons, the distribution of Aβ resembled both of these scenarios, with both punctate and non-punctate regions of Aβ immunoreactivity observed within the cytoplasm and nucleus (Figure 1C). When fluorescently tagged Aβ_1-40_ (10 µM) was applied to either wildtype or Tg2576 cortical neurons, we did not observe any difference in neuronal uptake or distrubtion of Aβ (results not shown).

### Intraneuronal Aβ increases the vulnerability of cortical neurons to neurotoxicity induced by extracellular Aβ

**Figure 2:**
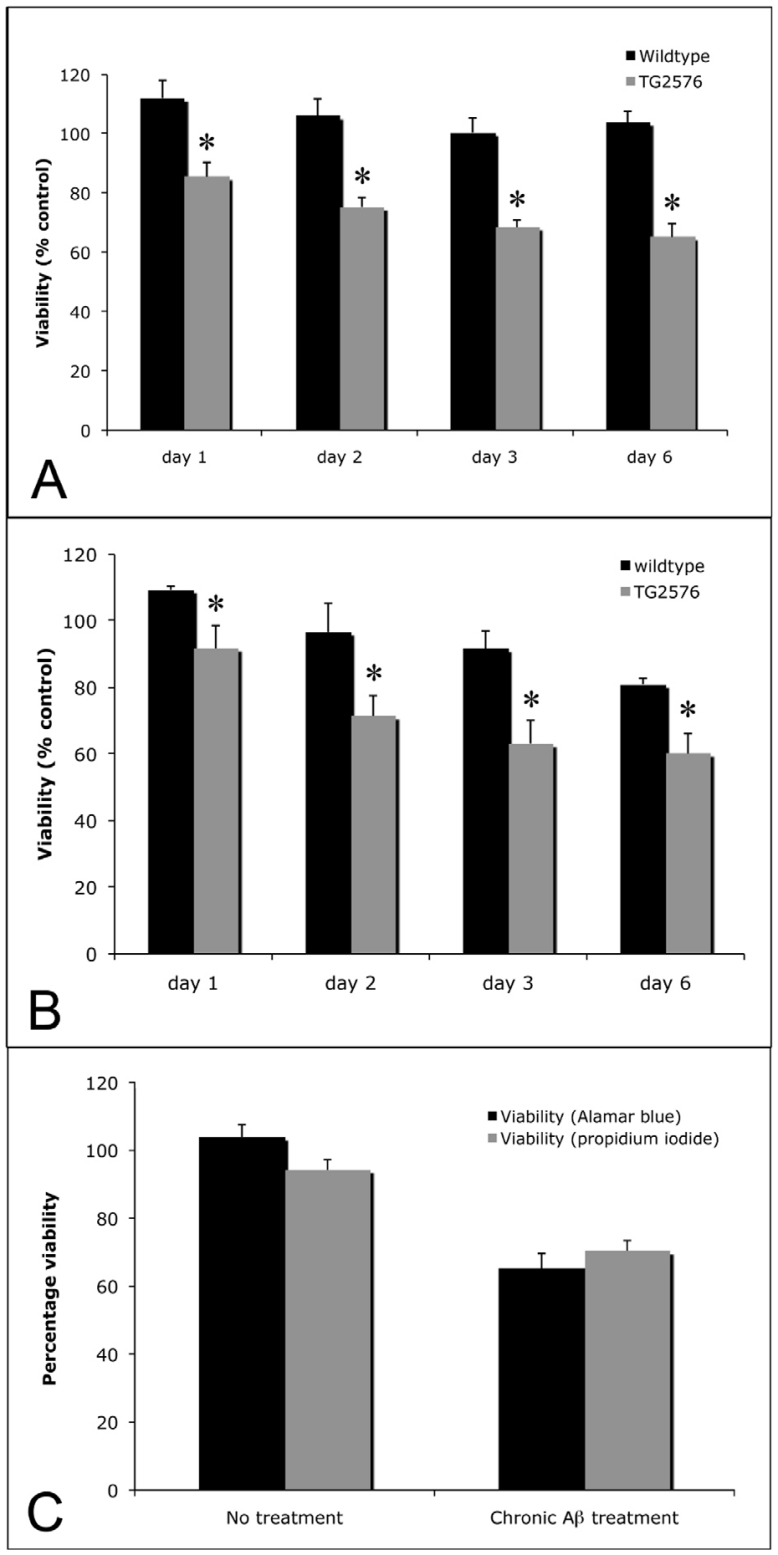
Tg2576 cortical neurons are more vulnerable to soluble Aβ-induced neurotoxicity. Wildtype and Tg2576 cortical neurons were treated daily with 1 µM (A) or 10 µM (B) of monomeric Aβ_1-40_ for 6 days, and neuronal viability (intracellular metabolism as assessed by Alamar Blue assay) assessed every 24 hours. At 1 µM concentrations, only Tg2576 neurons were vulnerable to Aβ_1-40_, resulting in approximately 30% cell death after 6 days (A). 10 µM Aβ_1-40_ was mildly toxic to wildtype neurons over the experimental timecourse, but killed 40% of Tg2576 neurons after 6 days (B). The Alamar Blue neurotoxicity assay produced similar results to direct counting of dying cells via propidium iodide uptake following treatment with 10 µM Aβ_1-40_ (C). * - p<0.05, ANOVA. Error bars represent standard error values from at least three replicates per experimental condition. This graph is representative of the results observed from 4 different experiments.

7DIV mouse cortical neurons (wildtype and Tg2576) were treated with soluble monomeric Aβ_1-40_ (1–10 µM) daily for a period of six days. Measurement of neuronal viability by an Alamar Blue assay revealed that while daily treatment with 1 µM Aβ_1-40_ was not toxic to wildtype cortical neurons at any point in the six day timecourse, this treatment resulted in a significant reduction in intracellular metabolism of Tg2576 neuronal cultures by approximately 30% after six days of continual Aβ_1-40_ treatment (Figure 2A). 10 µM Aβ_1-40_ was mildly neurotoxic to wildtype neurons, resulting in approximately 20% cell death after seven days of treatment (Figure 2B). However, Tg2576 neurons were far more susceptible to Aβ_1-40_ (Figure 2B). The neurotoxic actions of chronic exposure to Aβ_1-40_ upon Tg2576 cortical neurons were confirmed in propidium iodide uptake studies (Figure 2C), whereby only dying cells internalise and incorporate propodium iodide in the nucleus. These results suggest that the non-toxic accumulation of intraneuronal Aβ in Tg2576 cortical neurons increases their vulnerability to subsequent neurotoxicity induced by chronic exposure to normally sublethal (1–10 µM) levels of extracellular Aβ. Notably, in all cases when either wildtype or Tg2576 neurons were treated with vehicle alone, no change in viability was observed (results not shown).

**Figure 3:**
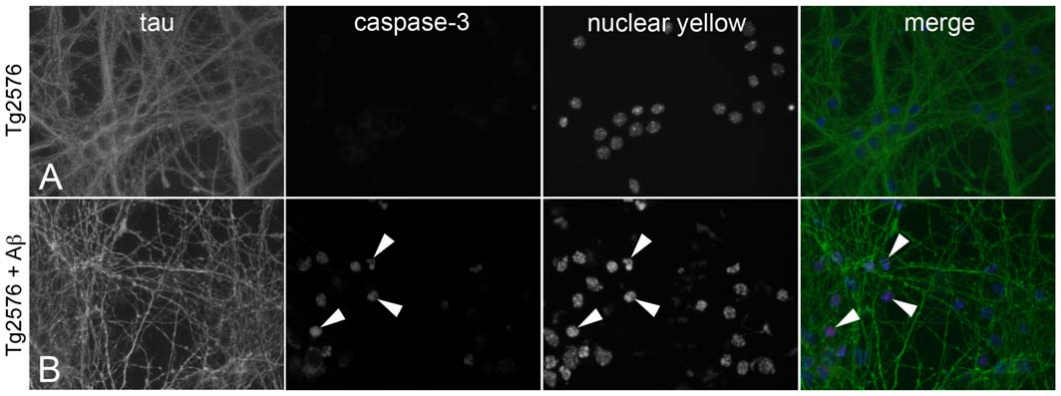
Soluble Aβ triggers caspase-3 expression in Tg2576 cortical neurons. Tg2576 neurons cultured for 14 days *in vitro* (DIV) showed no signs of caspase-3 activation (A). However, when 7 DIV Tg2576 neurons were treated with 1 µM Aβ_1-40_ daily for 6 days, a substantial number of neurons were found to express caspase-3 (B).

To investigate whether chronic exposure to both intra- and extracellular Aβ had initiated an apoptotic pathway of cell death, immunostaining for activated caspase-3 was performed. Almost no caspase-3 labelled cells were observed in 14 DIV Tg2576 neuronal cultures (Figure 3A). However, when 7 DIV Tg2576 neurons were treated with 1 µM Aβ_1-40_ for six consecutive days, caspase-3 immunoreactivity correlated with condensed or fragmented nuclei in approximately 30% of cells (Figure 3B), and direct counting found that the number of caspase-3 labelled neurons was equivalent to the number of neurons that incorporated propidium iodide (results not shown), indicating that the combination of both intra- and extraneuronal Aβ triggered an apoptotic pathway of neuronal death.

### Treatment with Aβ causes K^+^ flux-dependent neurotoxicity in Tg2576 neurons

**Figure 4:**
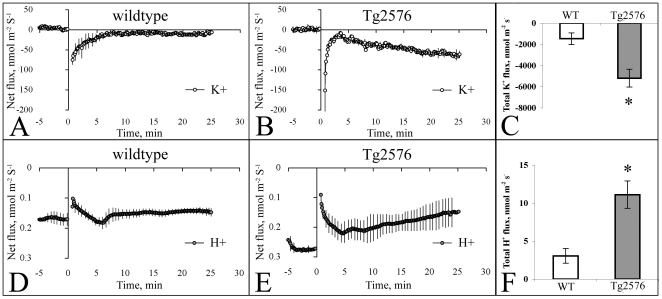
Soluble Aβ induces rapid efflux of K^+^ in Tg2576 cortical neurons. Treatment with 1 µM Aβ_1-40_ triggered rapid efflux of K^+^ from wildtype neurons (A), which returned to homeostasis within 10 minutes. However, Tg2576 neurons treated with Aβ_1-40_ displayed a continual efflux of K^+^ over the recording period (B). Measurement of total K^+^ flux over 25 minutes following Aβ_1-40_ treatment revealed that significantly more potassium was extruded from Tg2576 neurons than wildtype neurons (C). Aβ_1-40_ treatment caused a rapid influx of H^+^ in both wildtype (D) and Tg2576 (E) neurons, which stabilised within 5 minutes. However, from about 10 minutes post-treatment, Tg2576 neurons underwent a slow gradual influx of H^+^ (E). Measurement of total H^+^ flux over 25 minutes revealed that Aβ_1-40_ induced significantly greater influx of H^+^ into Tg2576 neurons in comparison to wildtype neurons (F). * - p<0.05, t-test. Error bars represent standard error values.

**Figure 5:**
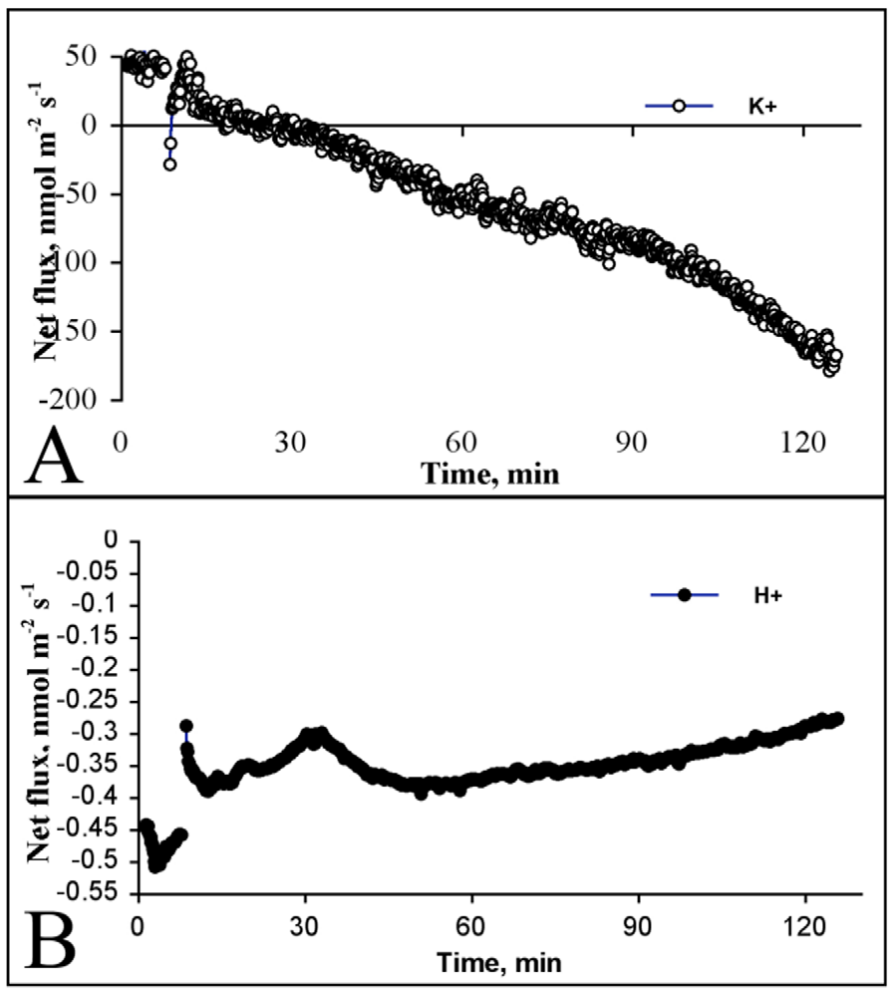
Soluble Aβ induces prolonged efflux of K^+^ in Tg2576 cortical neurons. Treatment with 1 µM Aβ_1-40_ triggered rapid efflux of K^+^ from Tg2576 neurons (A), which continued over the 120 minutes of recording. Aβ_1-40_ treatment caused a rapid influx of H^+^ in Tg2576 neurons (B), which did not stabilise over the 120 minute recording period.

There are a number of reports suggesting that extracellular Aβ triggers changes in ionic homeostasis of neurons, and that these changes contribute directly to neurotoxicity. To investigate whether intracellular Aβ alters the ability of neurons to maintain ionic homeostasis following extracellular Aβ treatment, we used a novel non-invasive microelectrode ion flux (MIFE) measuring technique. Using the MIFE approach, we directly observed that Aβ treatment triggered rapid efflux of K^+^ from wildtype neurons (Figure 4A), which returned to homeostasis within 10 minutes after Aβ_1-40_ treatment. However, K^+^ flux in Tg2576 neurons treated with Aβ_1-40_ did not return to homeostasis (Figure 4B). Instead, transgenic neurons exhibited a continual efflux of potassium for more than 120 minutes after Aβ_1-40_ treatment (Figure 5A). Measurement of total K^+^ flux over 25 minutes following Aβ_1-40_ treatment revealed that significantly more potassium was extruded from Tg2576 neurons than wildtype neurons (Figure 4C). Interestingly, continuous treatment of wildtype neurons for three days with 1 µM Aβ_1-40_ did not alter their ability to maintain K^+^ homeostasis following Aβ treatment (results not shown).

Concurrently, we also measured H^+^ flux of neurons in response to Aβ_1-40_ treatment. Wildtype neurons treated with Aβ_1-40_ demonstrated a rapid influx of H^+^, which stabilised within 5 minutes and remained stable for the entire recording period (Figure 4D). Tg2576 neurons displayed a similar response to Aβ_1-40_ (Figure 4E). However, from about 10 minutes post-treatment, Tg2576 neurons underwent a slow gradual influx of H^+^ (Figure 4E). The latter was further increased during the next 120 min post-treatment with Aβ_1-40_ (Figure 5B). Measurement of total H^+^ flux over 25 minutes after Aβ treatment revealed that Aβ_1-40_ induced significantly greater influx of H^+^ into Tg2576 neurons in comparison to wildtype neurons (Figure 4F).

It is generally accepted that excessive potassium efflux is a key early step in apoptosis. To confirm that prolonged extrusion of K^+^ is responsible for Aβ-induced neurotoxicity in Tg2576 neurons, cells were pre-treated with the classic K^+^ channel blocker 4-aminopyradine (4-AP) prior to Aβ treatment. This resulted in a significant reduction of Aβ-induced neuronal death in Tg2576 neurons (Table 1).

**Table 1:** Tg2576 cortical neurons are sensitive to soluble Aβ in a K^+^-dependent manner.

Treatment	Viability (relative to vehicle)
vehicle	100±6.9
1 µM Aβ	60.1±5.8*
1 µM Aβ + 2 mM 4-AP	87.4±6.0*^#^

Tg2576 cortical neurons were treated daily with either vehicle (PBS) or 1 µM of monomeric Aβ_1-40_ for 6 days, and neuronal viability assessed 24 hours after the final treatment. This resulted in a reduction in neuronal viability to approximately 60% of vehicle controls (* - p<0.01, student's t-test). However, when Tg2576 neurons were pre-treated with 2 mM 4-AP for 30 minutes prior to each Aβ_1-40_ treatment, this significantly reduced the neurotoxic action of exogenous Aβ_1-40_.

(^#^ - p<0.01, student's t-test).

### The presence of both intra- and extracellular Aβ causes the formation of axonal swellings in cultured neurons

**Figure 6:**
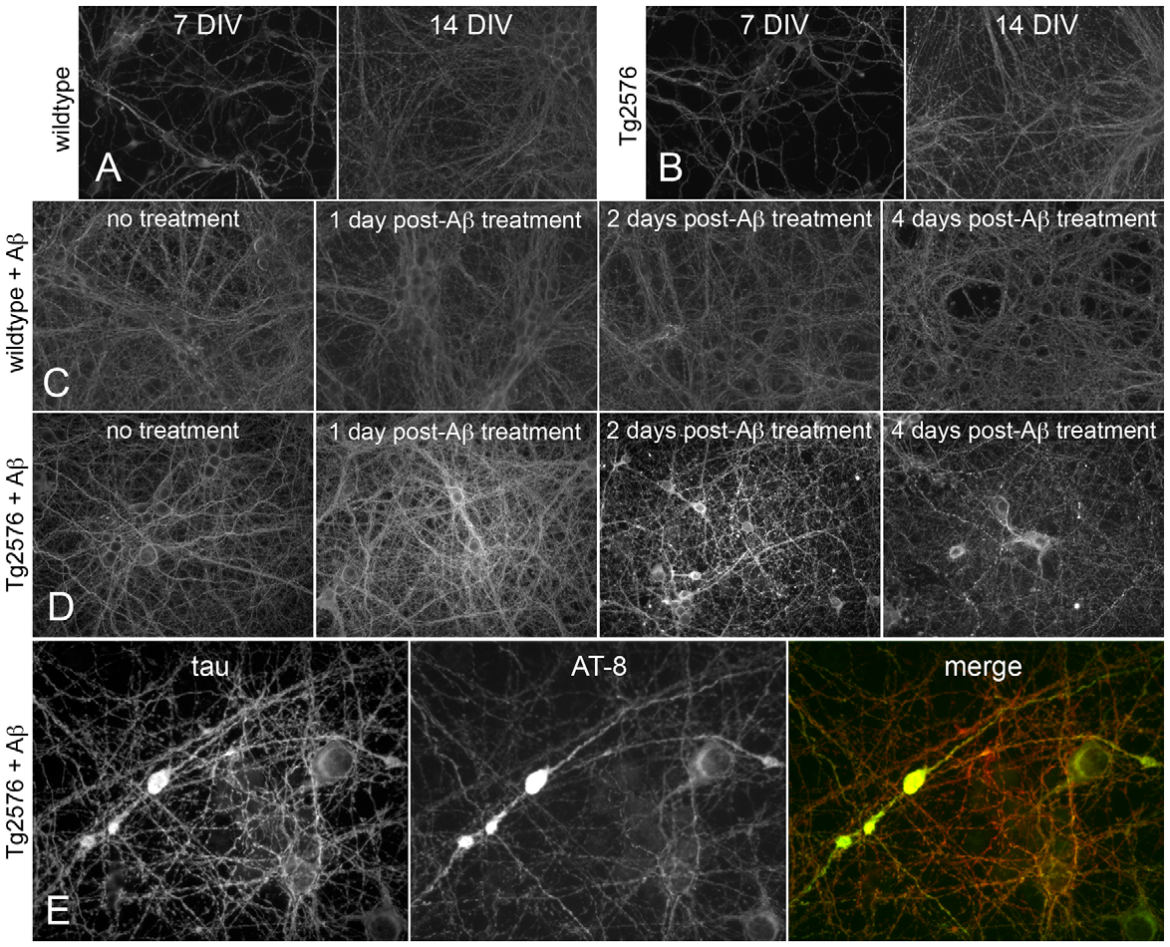
Soluble Aβ causes disruptions in tau distribution in Tg2576 cortical neurons. Tau immunolabelling of the axonal cytoskeleton demonstrated that axonal morphology was similar between wildtype (A) and Tg2576 (B) cortical neurons over 7–14 DIV. Six daily 1 µM Aβ_1-40_ treatments of 7DIV wildtype neurons had no discernible effect upon axonal morphology (C). However, substantial changes in tau-labelling were observed in Aβ_1-40_ treated Tg2576 neurons (D); including increased intensity of tau immunostaining after 24 hours, followed by blebbing and axonal fragmentation which worsened after four days of treatment (D). Furthermore, after four consecutive days of 1 µM Aβ_1-40_ treatment a number of axonal swellings, with dense accumulations of hyperphosphorylated tau, were observed in Tg2576 neuron cultures (E). scale bars  = 30 µm (A–D), 15 µm (E).

Immunolabelling of the axonal cytoskeleton (tau) revealed that there was no discernible difference in axonal morphology between wildtype (Figure 6A) and Tg2576 cortical neurons (Figure 6B) over 7-14 DIV. Daily treatment of 7DIV wildtype neurons with 1 µM Aβ_1-40_ over the six day experimental timecourse did not markedly alter axonal morphology (Figure 6C). However, substantial changes in tau-labelling were observed in Aβ_1-40_ treated Tg2576 neurons. Treatment of Tg2576 neurons with 1 µM Aβ_1-40_ resulted in a substantial increase in intensity of tau immunostaining after 24 hours (Figure 6D). After two daily treatments of Tg2576 neurons with 1 µM Aβ_1-40_, blebbing and axonal fragmentation was apparent (Figure 6D), which worsened after four continuous days of Aβ_1-40_ treatment (Figure 6D). Furthermore, after four consecutive days of 1 µM Aβ_1-40_ treatment, a number of axonal swellings were observed in Tg2576 neuron cultures (Figure 6E), but never in wildtype neurons treated with 1 µM Aβ_1-40_ for the same period of time (results not shown). These intra-axonal swellings were highlighted by dense accumulations of the microtubule-associated protein tau, including dense accumulations of hyperphosphorylated (AT-8 immunoreactive) tau (Figure 6E).

## Discussion

AD is a progressive neurodegenerative disease, in which neurons are likely to be exposed to sublethal concentrations of both intracellular and extracellular Aβ for extended periods of time. To experimentally model this situation, Tg2576 neurons (which accumulate substantial amounts of intracellular Aβ) and wildtype neurons received daily treatment with soluble, monomeric Aβ for 6 days. While this chronic exposure to Aβ did not kill wildtype neurons, it caused substantial apoptosis of Tg2576 neurons. Further studies revealed that Tg2576 neurons were unable to maintain K^+^ and H^+^ homeostasis following Aβ treatment, leading to prolonged extrusion of potassium and influx of protons into Tg2576 neurons. Furthermore, chronic exposure to 1 µM Aβ for six days caused the generation of hyperphosphorylated tau-immunoreactive axonal swellings in Tg2576 but not wildtype neurons. In summary, our data suggest that chronic exposure to sublethal levels of both intra- and extra-cellular Aβ induces neurodegenerative changes in cultured neurons that bear similarities to pathological hallmarks observed in AD. These changes appear to be driven by an inability of Tg2576 to maintain normal K^+^ homeostasis in response to continual exposure to extracellular Aβ.

The mechanism by which Aβ causes neurotoxicity or neuronal dysfunction remains to be fully resolved. Numerous studies have used cultured neurons to investigate the neurotoxic actions of Aβ, and can generally be classified into two paradigms; experiments whereby Aβ is applied acutely or chronically to cultured neurons (testing the effect of extracellular Aβ upon neurons), and experiments using neurons cultured from transgenic AD mice which express human Aβ (to test the effect of intracellular Aβ upon neurons). The former experiments have been particularly informative, revealing important information regarding the concentration and biochemical form of extracellular Aβ that exhibits toxicity upon cultured neurons; from such studies it is proposed that soluble oligomeric forms of Aβ at >5 µM concentrations are the most toxic form of extracellular Aβ to neurons [3]. In a similar manner, experiments using neurons cultured from transgenic AD mice have reported that intraneuronal Aβ increases the vulnerability of neurons to stressful cellular environments such as excitotoxicity and oxidative stress [11]. In some cases, the intraneuronal expression of Aβ itself can trigger apoptotis of neurons via a p53-dependent mechanism [21], [14]. It is worth noting that one simplistic manner in which exogenous Aβ may induce neurodegenerative changes in Tg2576 neurons is due to uptake of Aβ via LRP1 [16], which increases intracellular Aβ levels above a threshold level leading to decreased viability and alterations in tau distribution and phosphorylation. However, two pieces of evidence argue against this possibility. Firstly, the distribution of endogenous Aβ and internalised Aβ in Tg2576 is quite different, suggesting that these two pools of Aβ are not able to act in the same manner. And secondly, our MIFE studies demonstrate rapid and direct changes in ionic homeostasis of Tg2576 neurons triggered by application of exogenous Aβ, indicating that exogenously applied Aβ is probably acting in a different manner to intracellular endogenous Aβ.

While the studies discussed above have provided important information regarding the effect of extracellular and intracellular Aβ upon cultured neurons, it is important to consider that AD is a progressive condition, in which neurons are likely to be continuously exposed to sublethal concentrations of both intracellular and extracellular Aβ. To more accurately model this situation, Tg2576 neurons (which accumulate Aβ intraneuronally) were cultured in the presence of extracellular Aβ. This combination of intra- and extracellular Aβ induced caspase-3 dependent apoptosis of Tg2576 but not wildtype neurons. To elucidate the mechanisms underlying this, we used a non-invasive MIFE approach to observe changes in net ion flux of K^+^ and H^+^ ions in response to Aβ. Using this approach, we were able to continuously observe net ion flux of K^+^ and H^+^ for more than 2 hours. We found that Aβ treatment of wildtype neurons caused an immediate efflux of K^+^, which gradually returned to homeostasis within 10 minutes. However, we found that Aβ-treated Tg2576 neurons were unable to maintain K^+^ homeostasis, leading to prolonged leakage of K^+^ out of neurons. K^+^ efflux from cells is a key early initiator of apoptosis, as a low potassium intracellular microenvironment assists apoptosome formation and the activation of caspases and endonucleases. This suggests that the prolonged extrusion of K^+^ from Aβ-treated Tg2576 neurons was the cause of apoptosis in this study. This was confirmed by undertaking the same experiments in the presence of the K+ channel blocker 4-AP, which provided significant protection against Aβ-induced toxicity in Tg2576 neurons.

A novel element to the MIFE approach in this application is that it allows measurement of total flux of K^+^ into- or out- of cells, rather than the flow of K+ through particular channels/transporters that is observed through patch-clamp recording (electrogenic transporters) and pharmacological inhibitor studies. Our observations are in accordance with previous studies demonstrating that the neurotoxicity elicited by soluble Aβ upon neurons involves elevated K^+^ efflux, mediated through multiple pathways including enhanced activity of voltage-gated potassium channels [22], [23] and the Na^+^/K^+^ ATPase [24], [25]. Furthermore, increasing the extracellular K^+^ level to prevent K^+^ loss is also able to block Aβ-induced neuronal apoptosis [26]. We now demonstrate the direct measurement of net K^+^ flux of neurons in response to Aβ, and report that simultaneous exposure to both intra- and extracellular Aβ significantly impairs the ability of neurons to regulate K^+^ homeostasis. Given that intracellular accumulation of Aβ within neurons in the AD brain is only observed later in life, our data provides a potential mechanism that could explain why neurons in the AD brain become vulnerable to apoptosis later in life despite being continuously exposed to extracellular Aβ for many years.

While the toxicity of Aβ has been extensively studied in cultured neurons, two considerable limitations of these approaches have been that they have often involved the use of relatively immature neuronal phenotypes (cultured for between 1–4 days *in vitro*), and a treatment period of up to 24 hours. In this study we have cultured neurons at relatively high density for seven days *in vitro* prior to experimentation. Under these conditions, neuronal cultures contained dense networks of processes more representative of mature neurons. Chronic exposure of wildtype neurons to sublethal concentrations of Aβ did not alter axonal morphology of cultured neurons. However, Tg2576 neurons continuously treated with Aβ displayed substantial axonal pathology, including increased intensity of tau immunolabelling, axonal fragmentation and degeneration and the formation of axonal swellings that were packed with hyperphosphorylated tau. While it is well described that Aβ treatment of cultured neurons can directly cause tau hyperphosphorylation within 24 hours [27], our study is the first that we are aware of that demonstrates that Aβ can cause the generation of hyperphosphorylated tau-immunoreactive axonal swellings in mature neuronal cultures. Notably, these axonal swellings took four days to develop, suggesting that they represent a slowly evolving, secondary phase of Aβ-induced neurodegeneration. These dystrophic axonal manifestations resemble some of the key neuritic pathologies observed in the AD brain, suggesting that the prolonged effect of intra- and extracellular Aβ exposure upon neurons is a critical step in the neurodegenerative process underlying AD.

In summary, we propose that in the AD brain, intraneuronal accumulation of Aβ increases the vulnerability of neurons to subsequent chronic exposure to soluble Aβ. This combined exposure to intra- and extracellular Aβ leads to degenerative changes in neurons (such as axonal swelling and fragmentation) and apoptosis through a K^+^ efflux mediated mechanism.

## Methods

### Ethics Statement

All animal experimentation was performed under the guidelines stipulated by the University of Tasmania Animal Ethics Committee, which is in accordance with the Australian code of practice for the care and use of animals for scientific purposes.

### Cortical neuron cultures from Tg2576 and wildtype mice

Hemizygous Tg2576 male mice on a hybrid B6SjL background (Taconic) were crossed with wildtype B6SjLF1 females, and cortical tissue was removed from individual embryos from the pregnant wildtype mice (the embryos are either transgenic or wildtype), and cortical neurons isolated as we have described previously [28]. Cortical neurons from individual pups were maintained in Neurobasal medium (Gibco) containing 10% fetal calf serum, at 37°C in humidified air containing 5% CO_2_. The culture medium was replaced with serum- and glutamic acid-free culture medium after 24 hours, followed by half media changes twice weekly. To test the affect of chronic Aβ treatment upon cortical neurons, 1 µM or 10 µM of soluble monomeric Aβ_1-40_ was added exogenously to 7DIV cultured neurons daily for 6 days with assessment of cellular viability conducted each day. Lyophilised Aβ_1-40_ peptide was purchased from EZBiolab, and solubilised in sterile MQ water (which served as the vehicle control for all experiments). The Aβ_1-40_ peptide was used in this study because in our experience the Aβ_1-42_ peptide induces substantial acute neurotoxicity at 10 µM concentrations, making this peptide unsuitable for long-term studies. Note that each day, the entire culture medium was replaced with fresh media to which Aβ_1-40_ was added. All experiments were performed without knowing the genotype identity (wildtype or transgenic) of the individual neuron cultures. We generally obtained cultures from 10 embryos per pregnant animal, and they were usually split evenly between transgenic and wildtype. At the conclusion of each experiment, immunocytochemical detection of Aβ (using the 6E10 antibody, see details below) was used to genotype the cultures and reveal their identity for data analysis and interpretation. Untreated neuronal cultures at 14 days *in vitro* were used for this purpose, and neurons could be easily distinguished as containing either high- or low- levels of Aβ.

### Alamar blue viability assay

Neuronal viability was measured by the degree of cellular metabolic reduction of Alamar Blue®, as we have reported previously [29], [30]. Briefly, viability was determined by determination of the fluorescence of Alamar Blue in culture wells (excitation 535 nm, emission 595 nm), and was expressed as the percentage of the signal obtained from the vehicle-treated culture. Alamar Blue was applied at a 1∶10 dilution in culture media for 30 minutes, after which time it was collected and fluorescence measured on a fluorescent plate reader (Tecan Genios). In experiments involving repeated measurements of viability over a consecutive series of days, following Alamar Blue collection fresh media was applied to the neurons and the viability assay procedure repeated each day. Alamar Blue is non-toxic, and we have not observed any decline in neuronal viability when using this technique on consecutive days for up to one week (results not shown).

### Immunocytochemical labelling of neurons

At the completion of experiments, cells were fixed with 4% paraformaldehyde for 20 minutes and an antibody diluent containing 0.03% Triton-X detergent was applied. For immunocytochemistry, rabbit anti-tau (1∶5000; DAKO), mouse anti-Aβ (6E10; 1∶1000; DAKO) and mouse anti-hyperphosphorylated tau (AT-8; 1∶1000; Chemicon) antibodies were applied, and detected with appropriate Alexa-Fluor-488 or -594 conjugated secondary antibodies at a 1∶1000 concentration (Molecular Probes).

### Non-invasive microelectrode ion flux (MIFE) measurements in cultured neurons

The theory of MIFE measurements was reviewed recently [31] and the complete experimental procedure including ion-selective microelectrode fabrication [32] and neuronal culture preparation, immobilisation and recording were performed as described previously [30], [33]. Briefly, ion selective microelectrodes were silanised and filled with commercially available ionophore cocktails (Fluke catalog no. 60031 and 95297 for for K^+^ and H^+^, respectively). Microelectrodes for MIFE measurements were prepared on a daily basis and calibrated before and after measurements in a range of K^+^ and H^+^ concentrations. Cortical neurons for the MIFE measurements were grown for six days at a density of 1×10^5^ cells/well on poly-L-lysine-coated coverslips and chronically treated with soluble monomeric Aβ_1-40_ as described above. A cover slip with neural cells was washed in and adapted to the MIFE artificial CSF (aCSF) for one hour prior to experiments. The composition of the aCSF was: 150 mM NaCl, 0.5 mM KCl, 0.5 mM CaCl_2_, 1.5 mM MgCl_2_, 1.25 mM NaH_2_PO_4_, 5 mM NaHCO_3_, 25 mM glucose, pH 7.4. Electrodes were co-focused and positioned ∼5 µm above the neuronal monolayer and moved up and down by a computer-controlled stepper motor providing a travel range between 5 and 50 µm from the cell surface at a frequency of 0.1 Hz. After recording steady state flux for ∼5 min, neurons were treated with Aß and data acquired at a rate of 15 samples/sec and later averaged over 8 second intervals. For all ion flux measurements, the sign convention is ‘influx positive’ for a cation. The data were analysed using MIFE software with ion fluxes expressed in nmol m^−2^ s^−1^. A total flux was calculated as area between flux curve over the indicated experimental timeframe (25 min) and the starting flux value.

### Statistical analysis

For each experiment unless otherwise stated, a minimum of four wells from at least three separate cultures (derived from different animals), were used for quantification. Statistical analysis was completed using SPSS 16.0 (SPSS). When data was unequally distributed, data was transformed so that the residuals were approximately normally distributed. Statistical significance was calculated using t-test when only two experimental samples were compared, and One-Way ANOVA with Tukey's Post Hoc Test when multiple samples were compared. All graphical data is presented as mean ± SEM, significance p<0.05.
